# Migration, Urbanism and Health: Moving Toward Systems-Informed Policy and Practice

**DOI:** 10.12688/f1000research.158252.1

**Published:** 2024-11-08

**Authors:** Palmira Immordino, Rita Sà Machado, Sally Hargreaves, Furio Honsell, Karen Lau, Stefania Pascut, Irene Torres, Yang Xiao, Anna Ziersch, Cathy Zimmerman

**Affiliations:** 1 Health and Migration Department, World Health Organization, Switzerland and Department of Health Promotion, Mother and Child Care, Internal Medicine and Medical Specialties, University of Palermo, Italy, Palermo, Italy; 2 Health and Migration Department, World Health Organization, Switzerland and Directorate General of Health, Portugal., Lisbon, Portugal; 3 The Migrant Health Research Group, St George's, University of London, United Kingdom and the Consortium for Migrant Worker Health., London, UK; 4 Regional Assembly of Friuli Venezia Giulia Autonomous Region and University of Udine, Italy, Udine, Italy; 5 London School of Hygiene and Tropical Medicine, United Kingdom and the Consortium for Migrant Worker Health, London, UK; 6 WHO Healthy Cities Coordinator Udine Municipality, Udine, Italy; 7 Fundacion Octaedro, Ecuador; Inter-American Institute for Global Change Research, Uruguay, Montevideo, Uruguay; 8 College of Architecture and Urban Planning Tongji University, China, Shanghai, China; 9 Flinders Health and Medical Research Institute, College of Medicine and Public Health, Adelaide, Flinders University, Australia, Adelaide, Australia

**Keywords:** Migrant health; Migration and displacement; Urban policy and migration; Health determinants; Public health; Migrants; Refugees

## Abstract

Migration and displacement are pivotal determinants of urban health, influencing both direct and indirect health outcomes. Migrants may face unique health risks, often exacerbated by economic, social, and environmental factors encountered during transit or upon resettlement. As migration patterns shift due to geopolitical, climatic, and economic pressures, they reshape the global and urban policy landscapes in unpredictable ways, presenting challenges that will continue to evolve in the coming decades.

Current legal frameworks, in many cases, do not adequately account for migrant populations, hindering policy responses and complicating the protection of migrant health. Therefore, effective urban health interventions must be inclusive of migrant populations and expand beyond healthcare services. A systems-thinking approach that recognizes the broader determinants of health—including housing, employment, social services, and urban infrastructure—is essential to address the intersecting challenges migrants face.

Despite these challenges, migration remains crucial to the functioning of urban environments. Migrant workers consistently contribute to the healthy operation of cities, underpinning key infrastructure and services. However, to optimize policy responses and improve urban health outcomes, more robust data and evidence on the health risks and outcomes of migrants, as well as the structural drivers of migration, are needed.

Moreover, macro factors such as climate change, future pandemics, and geopolitical shifts are likely to influence both migration dynamics and migrant health. To anticipate and respond to these evolving challenges, a systems-informed and holistic vision of urban health is required—one that integrates migration into the broader urban policy and planning frameworks to foster healthier, more resilient cities.

## Content sections

1. Key messages
2. Introduction
3. Findings
4. Priority data and research

## 1. Key messages

- Migration and displacement are key determinants of urban health, both via direct exposures to health risks and as mediated by social or economic factors experienced by those on the move.
- Migration and displacement are changing the global and urban policy landscape in significant and unpredictable ways and will continue to do so in coming decades.
- Pre-existing legal frameworks often do not account for migrant populations, complicating policy responses to ongoing migration.
- Migrant-inclusive urban health interventions must go beyond health services and address the multiple, wider, intersecting systems that influence health risks and outcomes.
- Migration and migrant workers consistently contribute to the healthy functioning of cities and urban infrastructures.
- Responding to the challenges and opportunities posed by migration requires more robust evidence on migrant health risks and outcomes and the structural drivers of population mobility.
- A systems-informed, holistic vision of how migration interacts with other urban systems is fundamental to achieving the best urban health outcomes.
- Climate change, future pandemics, and geopolitics are among the macro factors that shape migration dynamics and migrant health outcomes; more work is needed to understand short and longer-term future impacts.

## 2. Introduction

Migration and displacement have become increasingly salient issues in the global health landscape. The vulnerabilities and opportunities associated with mobility and urbanization need to be better addressed and accounted for, particularly given that migrants and displaced people represent a heterogeneous group with varied circumstances, experiences and contributions.

Box 1. **Overview of migration terms used in the paper**

**Asylum seeker.** A person who is seeking international protection. ^1^ Prior to being granted legal status in the destination country, refugees are termed asylum seekers. Not all asylum seekers will be granted refugee status.

**Refugee.** According to the 1951 United Nations Convention and its 1967 Protocol Relating to the Status of Refugees, under international law and UNHCR’s mandate, refugees are individuals living outside their countries of origin who are in need of international protection because of feared persecution, or a serious threat to their life, physical integrity or freedom in their country of origin as a result of persecution, armed conflict, violence or serious public disorder. ^2^

**Migrant.** According to the IOM, a migrant is an “umbrella term, not defined under international law, reflecting the common lay understanding of a person who moves away from his or her place of usual residence, whether within a country or across a border, temporarily or permanently, and for a variety of reasons”. ^3^

**Internal migrant.** A person who has moved within internationally recognized state borders and includes rural-to-rural migration and rural-to-urban migration. ^4^

**lnternational migrant.** Defined by the United Nations Department of Economic and Social Affairs as any person who changes his or her country of usual residence. ^5^

Migration and displacement are key determinants of health, potentially affecting epidemiological patterns of chronic and infectious diseases, mental health issues, among others. Additionally, migrants and displaced persons may face barriers in accessing healthcare and other basic services (e.g., water, sanitation, housing, safe and nutritious food, safe and fair employment) due to language, cultural, or economic factors. They may have elevated risks of injury, either during the migration journey or because of unsafe living or working conditions at their destination country.

Migration to urban areas, whether between or within countries, is a defining issue for cities. Increasingly, urban health planning is considering migration patterns, migrant health characteristics and migrant-inclusive services. While global dialogue often focuses on international migration, internal migration, especially in low- and middle-income countries (LMICs), is more prevalent. Specifically, rural to urban migration is a modern feature of LMICs, particularly given the impending effects of climate change and loss of rural livelihood options.

This paper explores the relationships between migration and urban health and outlines potential implications for policy and practice. It examines how urban health advocates, practitioners and migrants themselves might respond to migration challenges and considers holistic approaches to urban health in the context of migration.

## 3. Urban health and migration: what do we know?

### 3.1 A snapshot of current migration in urban settings

The global movement of people, whether within borders or across them, is growing. As of 2020, worldwide estimates indicated that roughly 281 million individuals were international migrants, making up 3.6% of the global population. This number represented an increase of 128 million from 1990 and was over three times the estimated count in 1970. ^6^

Migration is determined by various determinants or drivers of individual and population mobility. The decision to migrate is influenced by circumstances in countries of origin, transit, and destination. Drivers of migration include economic, demographic, environmental, social, and political factors. People often move to improve their quality of life, access better opportunities for work or education, or escape conflict, violence, instability, persecution, or crises. Because urban centers are often seen as providing opportunities and better security, migration can contribute significantly to urban expansion.

The various types of migration contribute differently to urban growth and diversity in various contexts. International migration contributes to cultural diversity in high-income countries. Immigration adds to urban population growth, and in some contexts more so than growth from natural increase. Where both are high, as in some low-income countries, exceptionally high rates of urban growth are observed.

Frequently, migrants in megacities live in informal settlements and makeshift camps and, increasingly, intermediary cities also let unplanned settlements grow unchecked. Such living conditions place migrant populations at risk of poor health outcomes. More importantly, a lack of anticipatory planning, zoning, and building of infrastructure can lead to the costly and inefficient extension of services and more adequate shelter as an afterthought. ^7^ In addition, policy fragmentation across local and national levels may discourage health-oriented urban planning if city governments responsible for other essential services are not as accountable for health as are national health authorities.

Current estimates indicate that 169 million people are international migrant workers ^8^ – a figure that does not include the millions of internal migrant workers, nor refugees and forcibly displaced populations who *become* migrant workers in urban areas. And yet, extraordinarily little attention, and less action, focuses on migrant workers, despite figures that indicate that migrant workers are more than three times more likely than non–migrants to be in conditions of forced labour. ^9^ A systematic review found that compared with non-migrant workers, migrant workers were 45% less likely to use any health services. ^10^ Migrant workers, whether cross-border or internal migrants, are also over-represented in industries exposed to higher risk of occupational injury and fatality. ^11^

At least 10.1% of all international migrants are children, and many of them migrate unaccompanied or become separated from their parents. ^6^ Apart from sharing the vulnerabilities experienced by adults, children in urban centers are at an increased risk of, at minimum, homelessness, becoming exposed to hunger and violence, and being coerced into panhandling and micro-trafficking. ^12^ Although rural-urban migration is more common among younger people, growing numbers of people aged 60 years and older are moving to urban areas around the world. ^13^

### 3.2 Determinants of health in the context of migration and urban health

The Dahlgren and Whitehead model of the social determinants of health includes different factors that can affect and impact health – including individual, lifestyle, social and community factors and working and living conditions. ^13^ The WHO Commission on Social Determinants of Health (2008) requested that urban governance and planning explicitly address equity, as cities can concentrate threats to health.

Social and structural determinants of health substantially influence the health and the integration of migrant populations in urban settings. For example, state regulations that fail to provide migrants with basic services, including safe housing, social protection, and healthcare, set the foundation for poor health outcomes. Other structural issues, like the segregation of migrant communities in less desirable neighborhoods on the outskirts of many cities, contribute to a lack of access to services and generate new health risks, for example those associated with long commutes or neighborhood safety or environmental hazards.

Intersectional considerations often further increase risks for many migrants. For example, inequitable gender norms sustain isolation and abuses experienced by female migrants. Female migrants, especially asylum-seeking and refugee women and girls, are particularly vulnerable to physical and sexual violence ^14^ and early or forced marriage. ^15^ Migration-related discrimination can amplify pre-existing gender inequalities, leaving abuse survivors less able to access protection or services for sexual, reproductive, and mental health. Even where social protection is ensured, access to healthcare services may be influenced by migratory status in other ways. International migrants, especially those in marginalized situations, may face sociocultural challenges such as language barriers and lack of familiarity with the health system, ^16^ as well as a lack of culturally appropriate services.

In some countries, “health navigator” programs train and certify community health workers to provide free assistance to migrants in their own language. Mobile phone health tools can also support the interventions of community health workers ^17^ in countries with high mobile phone coverage, including by teaching the local language to improve health literacy. Community centers are also important health promoting sites, providing access to computers, internet, and other digital resources, for example. Here, migrants can access health information and communicate with government agencies and other organizations (including to schedule healthcare appointments). Such centers can also provide other services important to health such as safe spaces to socialize, play and engage in physical activity that may be scarce in urban environments. Ultimately, greater connectivity and community center-based engagement are also useful to encourage and support participation in the decision-making processes of government agencies and other organizations to better address the needs of migrant populations.

### 3.3 Integration and social cohesion: impact on health and well-being

Integration in host communities is a significant issue for many migrants, who often experience disadvantage relative to native residents in many aspects of daily life. ^18^ Migrant populations can encounter challenges with respect to adaptation and integration, including competition and conflict among people, groups, and cultures. Adjusting to a new urban lifestyle can mean supplementing, or at times replacing, old social support systems and cultures with new ones. Challenges like securing housing, finding work, and dealing with peer pressure from family and relatives can lead to heavy psychological burdens and resulting mental health challenges ^19^ like anxiety, despair, tension, low self-esteem, and loss of control. ^20^ Among the many factors affecting mental health, the social environment has been shown to have a significant impact. ^21^

Social cohesion, which describes the degree of comradery and solidarity within a community, ^22^ can be crucial in promoting the mental health and general wellbeing of migrants. Strong social cohesion can give people a sense of support and belonging, which can act as a protective barrier against the negative effects of trauma and displacement. Programs for linguistic and cultural orientation, community-building initiatives, and specialized mental health interventions are a few examples of supportive approaches to build cohesion. Social cohesion can affect migrants’ mental health through three general mechanisms. ^23^ First, it can encourage health-related activities and norms and exerts social control over deviant behaviors. Second, it can promote social organization, making it simpler for people to access health services. Third, it can influence psychosocial processes like offering emotional support, raising self-esteem, and fostering mutual respect. In addition, communities where people trust one another are more likely to offer assistance and support, which can help to promote mental health. ^24^

### 3.4 Migrant-inclusive urban health: A systems framework

To promote urban health that genuinely includes migrants, changing the functioning of the health system alone, while crucial, is insufficient. For instance, reducing high risks of road traffic fatalities or respiratory disease due to air pollution among migrant workers depends not only on including them in data collection by health information systems, design of appropriate health promotion information, and access to health services, but also on policy decisions within the transport, energy, and planning sectors, among others. And, vice versa: even if health promotion activities and health services are geared to include migrant workers, services may go unused without accurate data about which groups of workers are affected and how to reach specific groups, such as day laborers, who may be reluctant to lose wages to seek care.

**Figure 1.**
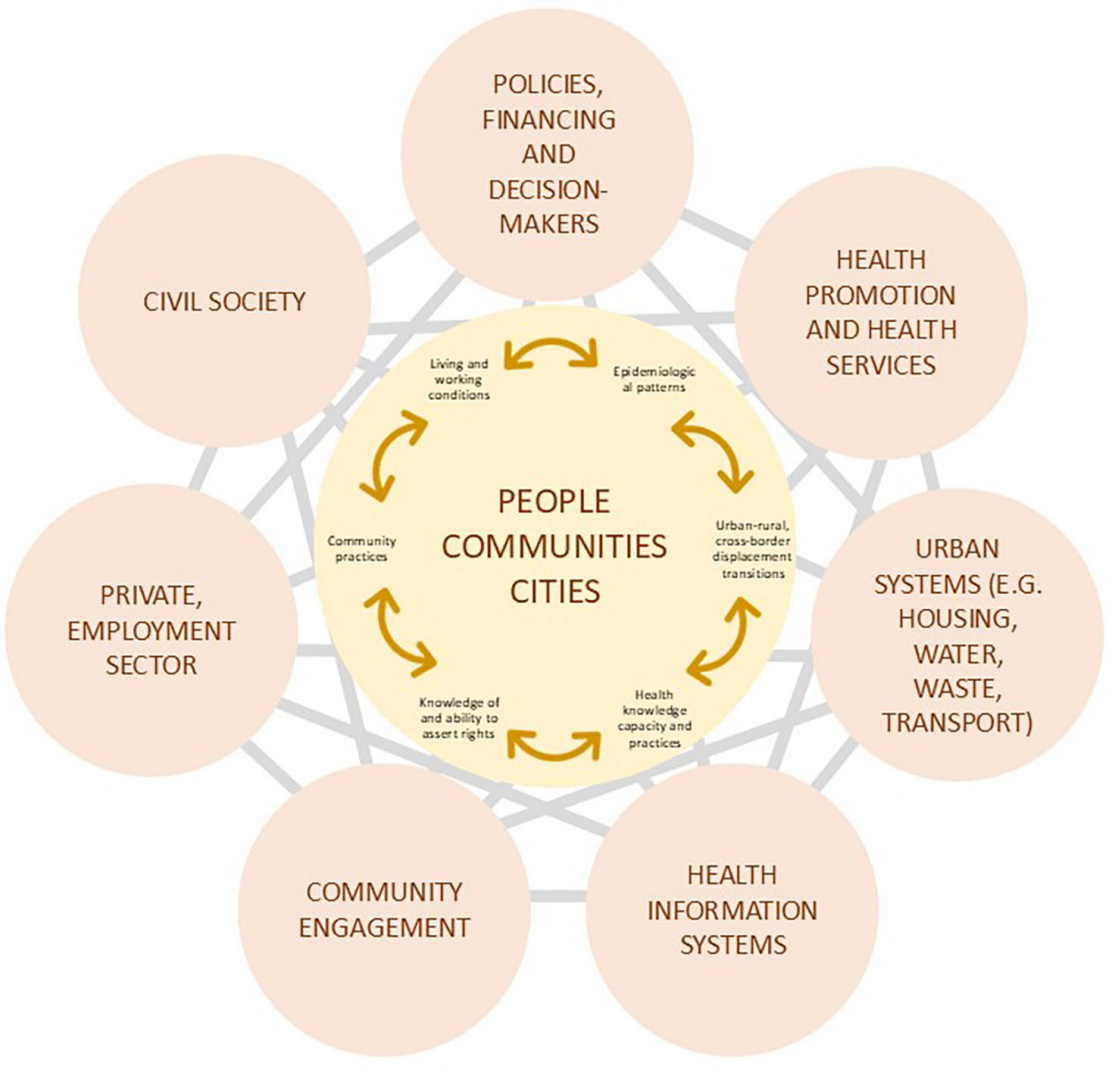
Systems-informed urban health response to migration-related health needs.

Figure 1 proposes a migrant-inclusive urban health framework to indicate various systems that might interact to affect the health of migrants. The outer spheres of Figure 1 represent multiple systems that can affect health beyond the health system alone. At the centre are “people”, “communities,” and “cities” to indicate that these systems behave in ways that affect individual migrants, migrant subgroups (as defined by nationality, legal status, work sector, etc.) and the health patterns of urban populations of migrants *and* non-migrants. These systems can also influence epidemiological risk and outcome patterns (e.g., infection and non-infectious diseases, nutrition, mental health) that include or exclude migrant groups and determine how well health information systems track disease patterns. Similarly, actions of multiple systems, such as immigration systems, health systems and employment practices, often combine to determine whether migrants are exposed to health hazards, whether they know their rights and entitlements and whether they work in safe and fairly paid jobs. Finally, civil society often fills in gaps where health systems or government services are not available for migrants.

The systems framework suggests that creating inclusive urban health will require going beyond health sector services, and addressing the important integration influences, such as social networks and community engagement, which can provide highly valued psycho-social support and help communicate health-related information. ^25^ Central to all these health-inducing factors are legal structures and budget allocation systems to support migrant health amidst increasing urbanization. Evidence suggest that these frameworks are crucial in ensuring migrant health: non-health-targeted public policies can significantly affect migrant health. ^26^ This underscores the need for a holistic approach to migrant health that considers the broader policy environment. Policies that promote social inclusion, provide easier access to healthcare, and support economic integration can significantly improve health outcomes for migrants.

A strategic and integrated approach to migration and urban health ^27^ must rely on the recognition that migration health is influenced by decisions made at various levels, from global to local, within and beyond the health sector. The ethical challenges in these decisions impact the provision and accessibility of healthcare services; it is crucial to consider specific ethical dimensions and vulnerabilities in each context. ^28^ Values such as solidarity, duty, equity, trust, and reciprocity, are central to addressing the health needs and rights of migrants.

Moreover, migration requires effective multilevel governance of migration-related issues at local, national, and international levels. At the local level, this means building alliances and partnerships, raising awareness in the population, and supporting the development of migrants’ potential in their new home. In many contexts, the spontaneous formation of numbers of local, voluntary associations and groups is quite common, driven by a moral duty of care. These can be extremely valuable because they develop detailed knowledge and respond quickly during emergencies. Local authorities need to support and involve such groups, especially where emergencies become prolonged. Because such groups often support undocumented migrants, on the edge of legality, they may be excluded from government action, yet this may set up serious tensions and conflicts within the local population.

To address this multiplicity of influences requires a holistic approach that encompasses all aspects of migrant health, including decent housing, access to basic services, social participation, decent work, emergency preparedness, and mutual support and social cohesion with native populations. A holistic approach transcends the strictly medical aspects of health care, recognizing the importance of a broader perspective for achieving migrant health in all aspects of their lives, and therefore public health for all. Promoting healthy lifestyles and prevention among migrants is also critical to long-term health trajectories, as exemplified by the long-term experiences of the *WHO Healthy Cities* project. ^29^

## 4. Data and research: what evidence is needed to promote urban health equity?

Strategic, integrated action to improve urban health and health equity in the context of migration requires accurate, efficient, and timely data that builds on the existing evidence base. Below we highlight priority areas for new research. In addition to those highlighted, the first WHO Global research agenda on health, migration and displacement outlines key global research priorities and acts as a framework and guide for prioritizing research in this area. ^30^ This framework can be used as a catalyst for further research in emerging areas such as urban health and migration, and to strengthen research activities and translation into policy and practice on the topic at regional and national level.

**Priority research areas**

**A local and global lens.** Detailed information on local urban settings is important for understanding and developing context-specific interventions to improve urban health and health equity in the context of migration. However, understanding global factors influencing the movement of people—particularly between and within low- and middle-income countries in the Global South, which host the largest numbers of migrants—is critical not only to global and regional action but for local interventions. Cross-country research on migration and urban health would supplement focused local studies by learning across two or more countries, e.g., using comparable administrative data, to examine key questions (see below).

**Population mobility.** Population mobility tracking, both between and within countries, will help understand how urban settings affect health and vice versa. Some countries have adopted dispersal policies for new arrivals, particularly for refugees and asylum seekers, seeking to deconcentrate migrant groups and to match them to available resources such as housing and health services. ^31^ Understanding the factors driving mobility within countries, including both push and pull factors, to and away from and within urban contexts, and circular and seasonal migration is important for urban health decision-making.

**Pandemics and other health stressors.** COVID-19 had a significant effect on the flow of migration between and within countries and threw a spotlight on urban health inequities, including the role of the built and social environment and health resourcing. Data collected over the pandemic about health impacts and inequities and examining this through a migration lens are important both in terms of learning about some of the drivers of health inequities and in terms of preparedness for future health events – e.g., decision making about distribution of health infrastructure and resources and consideration of specific requirements of migrants in any public health measures.

**Structural drivers.** Complex upstream and downstream determinants of health influence outcomes in urban settings and understanding the structural drivers of urban health inequities associated with migration will contribute to informed health decision-making. For example, policies of nation states vary regarding settlement of migrants, including refugees and asylum seekers. These policies include immigration laws (e.g., mandatory detention of asylum seekers arriving by boat in Australia or regional resettlement policies that direct new arrivals away from urban areas). Influential policies also include housing and industrial relations policies that intersect with immigration policies (e.g., lack of social housing, poor social security safety nets). The Health in All Policies approach, championed by the WHO, emphasizes that non-health sectors should consider health impacts. Examining, for example, how migration and other policies affect health would provide evidence to transform policy- related structural drivers. ^32^

**An intersectional approach.** In addition to structural drivers, migration related-health inequities in urban settings are also influenced by oppression and discrimination, such as racism, sexism, agism, ableism, and heteronormativity. ^33^ It is essential to understand how these systems impact health, both in the policies around migration and the day-to-day experiences of migrants in urban areas. For example, policies can affect housing or employment discrimination and inequitable resource distribution for health and other services that are targeted away from areas of high migration settlement.

**Two-way integration.** Integration is a two-way process of mutual accommodation between incoming and receiving communities and affects the health of migrants. However, research has tended to focus on the extent to which migrants are able to adapt to their new communities (e.g., securing work, housing, etc.), rather than how receiving communities may themselves adapt and change. ^34^ Further data are required in urban settings on attitudes towards accommodation of receiving communities as well as the effectiveness of interventions to improve receiving community social environments (e.g., the Welcoming Cities initiative in Australia that accredits local areas for their efforts to promote inclusion and diversity ^35^) and examination of the ways that employment or other systems evolve and adapt to respond to the needs of migrants.

**Political will.** More evidence is needed on the social and economic impacts of migration at the levels of cities and whole societies and on how different migration policies affect a range of societal goals. A better understanding of how to secure benefits and counter potential risks from migration will inform political debates and counter xenophobia, while opening the door to more effective approaches to urban health.

**Nature of approaches and types of data.** Advocacy for improved health equity in urban settings requires the centering of **lived experience**, both of incoming and receiving communities, making the voices of refugees and migrants heard through participatory research approaches. It is also crucial to have intersectional and interdisciplinary approaches to data collection and analysis in migration and urban health research ^36^ and to supplement and extend research through digital data. There are considerable opportunities to consider a range of questions regarding migration and urban health through data linkage approaches that enable existing datasets to be used to answer more comprehensive questions that span focus areas. Longitudinal research designs can answer questions about causation and time lags in relation to urban health and migration and might be considered in selected cases. Complex systems, ^37^ and implementation science can give a unique perspective particularly on upstream determinants of health in urban settings and might facilitate the uptake of evidence-based practice and research by practitioners and policy makers.

## 5. Conclusion

For urban health strategies to be effective, governments and other decision-makers must take account of the contemporary reality of population mobility and the fundamental contribution of migrants to the functioning of cities and urban health. To promote inclusive urban health responses, decision-makers must remain alert to practical health-promoting strategies that do not expect migrants to reach the services, but instead reach out to migrants where they live and work, including interventions such as outreach initiatives and mobile clinics, as well as ensuring that policy settings are reflective of people’s needs.

Recognizing the social determinants of health of migrants who may be exposed to multiple health risks, also means adopting systems science perspectives. ^38^ Systems science perspectives recognise the complexity of health influences and the interactions between health intervention opportunities. To promote urban health that includes mobile populations, strategies need to go beyond narrow ‘health systems’ approaches by harnessing the interactions between the many health-influencing systems (e.g., immigration, financial, social, political). Evidence repeatedly indicates that establishing good coordination between these multiple systems will help advance migrant-inclusive urban health responses. ^39^

Among the greatest risks to inclusive urban health in most settings are the political winds that fuel the fires of discrimination against diversity. Xenophobic and exclusionary rhetoric readily hinders the gears of those systems that could be including migrants in one-health strategies. Future urban health depends on building political will for change and on migrants and non-migrants joining together to design systems strategies that treat health as a mutual priority, because “there is no *they* but only *us*” *.*

Therefore, to promote urban health, it is fundamental to prioritize migrants’ health by ensuring policies that are built on evidence-informed decision-making.

## Ethics and consent

Ethics and consent not required for the performed study

## Data Availability

No data are associated with this article.
