# Supplementary material for: Migration, Urbanism and Health: Moving Toward Systems-Informed Policy and Practice
Source: F1000Res. 2025 Jun 18;13:1344. Originally published 2024 Nov 8. [Version 2] doi: 10.12688/f1000research.158252.2 (PMC12264471; doi:10.12688/f1000research.158252.2)
Supplement: Peer review history

# Reviewer report: Reviewer response for version 2

**DOI:** 10.5256/f1000research.183567.r395623

**Published:** 2025-07-30

**Authors:** AKM Ahsan Ullah

**Affiliations:** 1 Universiti Brunei Darussalam, Bandar Seri Begawan, Brunei

I have read the article *"Migration, Urbanism and Health: Moving Toward Systems-Informed Policy and Practice"* with interest. To my view, this is a good contribution to contemporary migration and public health scholarship. The authors address a growing gap in the intersection of urbanization, health equity, and human mobility by proposing a systems-informed, holistic policy framework. The article is relevant in the context of increasing global migration, climate-induced displacement, and urban health disparities in low- and middle-income countries (LMICs). Its emphasis on social determinants of health, two-way integration, and structural drivers of inequity aligns it with critical debates in global health justice, urban studies, and migration governance.

 The article is built upon a qualitative synthesis of policy documents, academic literature, and illustrative case references. The integration of the Dahlgren-Whitehead model and systems-thinking frameworks sound good to me. However, the paper could be strengthened by specifying criteria for literature inclusion, incorporating more empirical case studies, and clearly distinguishing between internal and international migration in urban settings. The theoretical framing is relevant and effectively explains the multifactorial nature of health inequities, although it could benefit from deeper engagement with intersectionality theory and decolonial urbanism literature.

 The revised version successfully incorporates most reviewer suggestions. The authors improved the abstract, clarified scope and terminology, expanded the discussion of systems-thinking, and added ethical reflections on data use. However, I see some issues the author can think about to better their work.

 First, the findings are conceptually rich but not empirically grounded.

 Second, although the conclusion is stronger in linking back to key messages, more actionable recommendations for specific stakeholders would improve its policy utility.

 Third, dominant literature on postcolonial urban health (e.g., Lawhon, Lancet Migration) is underused.

 Fourth, the paper still conflates types of migration (e.g., refugees, internal migrants) without sufficient analytical distinction.

 Fifth, despite highlighting participatory approaches, migrant voices are largely absent from the analysis.

Is the review written in accessible language?

Yes

Are all factual statements correct and adequately supported by citations?

Yes

Are the conclusions drawn appropriate in the context of the current research literature?

Partly

Is the topic of the review discussed comprehensively in the context of the current literature?

Partly

Reviewer Expertise:

https://fass.ubd.edu.bn/staff/profiles/ullah.html

I confirm that I have read this submission and believe that I have an appropriate level of expertise to confirm that it is of an acceptable scientific standard, however I have significant reservations, as outlined above.

# Reviewer report: Reviewer response for version 2

**DOI:** 10.5256/f1000research.183567.r393656

**Published:** 2025-07-15

**Authors:** Cheng Chow

**Affiliations:** 1 The University of Texas at Austin, Austin, USA

Thank you for the opportunity to review this article. This revised version represents a strong conceptual and policy-oriented contribution to the migration health literature. However, a few minor issues should be considered to enhance clarity and impact:

 It would be helpful to explicitly state that this is a conceptual synthesis informed by existing literature and policy documents. If possible, also consider specifying the LMIC focus earlier in the abstract to better align with the scope outlined in the introduction.

 While the paper is conceptually rich, it would benefit from a few illustrative empirical examples to demonstrate how the proposed systems framework has been or could be applied in real-world contexts. These would help to ground the recommendations and increase policy relevance.

 Although both international and internal migration are acknowledged, the manuscript could more clearly delineate how health challenges and governance responses vary across migration types (e.g., circular, seasonal, or protracted displacement). This would add nuance to the discussion and improve its utility for diverse policy audiences.

 The manuscript refers to itself as a “qualitative review,” but more detail would enhance transparency. I recommend briefly outlining the search strategies, inclusion criteria, or types of sources used (even informally). It would also be helpful to clarify whether the review is best characterized as a narrative synthesis, scoping review, or policy mapping exercise.

Is the review written in accessible language?

Yes

Are all factual statements correct and adequately supported by citations?

Partly

Are the conclusions drawn appropriate in the context of the current research literature?

Yes

Is the topic of the review discussed comprehensively in the context of the current literature?

Yes

Reviewer Expertise:

My research lies at the intersection of migration, health, and policy.

I confirm that I have read this submission and believe that I have an appropriate level of expertise to confirm that it is of an acceptable scientific standard, however I have significant reservations, as outlined above.

# Reviewer report: Reviewer response for version 2

**DOI:** 10.5256/f1000research.183567.r392969

**Published:** 2025-07-15

**Authors:** Johannes Bhanye, Ruvimbo Shayamunda

**Affiliations:** 1 African Climate and Development Initiative, Department of Environmental and Geographical Science, University of Cape Town, Rondebosch, Western Cape, South Africa; 2 Faculty of Natural and Agricultural Sciences, Urban and Regional Planning, University of the Free State, Bloemfontein, Free State, South Africa

The authors significantly improved their manuscript and I recommend for indexing.

Is the review written in accessible language?

Yes

Are all factual statements correct and adequately supported by citations?

Partly

Are the conclusions drawn appropriate in the context of the current research literature?

Yes

Is the topic of the review discussed comprehensively in the context of the current literature?

Yes

Reviewer Expertise:

Migration, urban studies, climate change

We confirm that we have read this submission and believe that we have an appropriate level of expertise to confirm that it is of an acceptable scientific standard.

# Reviewer report: Reviewer response for version 1

**DOI:** 10.5256/f1000research.173821.r373586

**Published:** 2025-05-29

**Authors:** Nadia Charania

**Affiliations:** 1 Auckland University of Technology, Auckland, Auckland, New Zealand

Thank you for the opportunity to review this important and timely piece that proposes a migrant-inclusive urban health framework drawing from systems-thinking and a holistic model of health. The manuscript is well-written, and the recommendations it presents are both practical and relevant for advancing health equity in urban contexts by drawing attention to the important role of migration. I believe this work will be of significant interest to many academics, practitioners, and policymakers. I only have a few minor suggestions for improvement, outlined below.  

- The *Abstract* reads well and introduces the broad context within which the presented arguments are made. One minor suggestion would be to make the aim and focus of the paper clearer, perhaps by adding a sentence similar to the last two sentences of the Introduction section.
- In section 3.1 *A snapshot of current migration in urban settings*, for the sentence that reads “Frequently, migrants in megacities live in informal settlements and makeshift camps and, increasingly, intermediary cities also let unplanned settlements grow unchecked” - it would be helpful to clarify which context (e.g., LMICs) this is referring to and include a supporting reference.
- In section 3.3 *Integration and social cohesion: impact on health and wellbeing*, for the sentence that reads “First, it can encourage health-related activities and norms and exerts social control over deviant behaviors.” It would be helpful to clarify what it meant by ‘deviant behaviors’.
  - Also, the authors may wish to highlight some successful examples thereby providing evidence for the participation and inclusion of migrant voices.

- In section 3.4 *Migrant-inclusive urban health: A systems framework* – it would be helpful to include more literature here about systems-thinking and the value of incorporating complex systems thinking into these areas.
- In section 4 *Data and research: what evidence is needed to promote urban health equity? *several priority research areas are proposed. It would be helpful to outline any potential unintended consequences and how to mitigate these. For instance, in relation to the proposed research area of population mobility, there may be some unintended consequences of such population mobility tracking.

Is the review written in accessible language?

Yes

Are all factual statements correct and adequately supported by citations?

Yes

Are the conclusions drawn appropriate in the context of the current research literature?

Yes

Is the topic of the review discussed comprehensively in the context of the current literature?

Yes

Reviewer Expertise:

global health, migration, health behaviours

I confirm that I have read this submission and believe that I have an appropriate level of expertise to confirm that it is of an acceptable scientific standard, however I have significant reservations, as outlined above.

# Reviewer report: Reviewer response for version 1

**DOI:** 10.5256/f1000research.173821.r345692

**Published:** 2024-12-31

**Authors:** Johannes Bhanye, Ruvimbo Shayamunda

**Affiliations:** 1 African Climate and Development Initiative, Department of Environmental and Geographical Science, University of Cape Town, Rondebosch, Western Cape, South Africa; 2 Faculty of Natural and Agricultural Sciences, Urban and Regional Planning, University of the Free State, Bloemfontein, Free State, South Africa

The paper makes significant contributions to understanding the complex relationship between migration, urbanism, and health. These suggestions can enhance the depth and clarity of the analysis, ensuring that it effectively communicates its findings and implications to an interdisciplinary audience of researchers, policymakers, and practitioners in the field of urban health.

-
  - Consider refining the abstract to include a brief mention of the methodological approach or specific case studies if any were used.
  - The introduction could benefit from a more detailed discussion on how urban health challenges are uniquely modified by migration, possibly by providing a few global examples.
  - Consider mentioning the scope of the paper early on, such as geographic focus, if applicable, or specific types of migration (e.g., rural-to-urban, international).
  - Also be specific about the type of migration. International?
  - The paper could be more impactful by incorporating case studies or examples that illustrate the key points. This would not only break down the theoretical content but also provide practical insights into how these challenges are managed in different urban settings.
  - Suggest a clearer outline of gaps in current research and how addressing these could inform better policy-making. This section would benefit from a critical analysis of the limitations in existing studies on migration and urban health.
  - It would be useful to propose specific methodologies for future research, such as longitudinal studies or mixed-methods approaches, to address the complex dynamics of migration and health.
  - The conclusion could be strengthened by explicitly linking back to the key messages introduced at the beginning and discussing how the findings support these messages.
  - Propose in the conclusion a few strategic recommendations for policymakers, urban planners, and health professionals on integrating migration considerations into urban health strategies.
  - Check for grammatical errors and syntax issues that could hinder the readability of the paper.

Is the review written in accessible language?

Yes

Are all factual statements correct and adequately supported by citations?

Partly

Are the conclusions drawn appropriate in the context of the current research literature?

Yes

Is the topic of the review discussed comprehensively in the context of the current literature?

Yes

Reviewer Expertise:

Migration, urban studies, climate change

We confirm that we have read this submission and believe that we have an appropriate level of expertise to confirm that it is of an acceptable scientific standard, however we have significant reservations, as outlined above.
